# Spectral Library-Based Single-Cell Proteomics Resolves Cellular Heterogeneity

**DOI:** 10.3390/cells11152450

**Published:** 2022-08-07

**Authors:** Lakmini Senavirathna, Cheng Ma, Ru Chen, Sheng Pan

**Affiliations:** 1 The Brown Foundation Institute of Molecular Medicine, University of Texas Health Science Center at Houston, Houston, TX 77030, USA; 2 Department of Medicine, Baylor College of Medicine, Houston, TX 77030, USA; 3 Department of Integrative Biology and Pharmacology, University of Texas Health Science Center at Houston, Houston, TX 77030, USA

**Keywords:** single-cell proteomics, proteomics, mass spectrometry, spectral library, cellular heterogeneity, systems biology

## Abstract

Dissecting the proteome of cell types and states at single-cell resolution, while being highly challenging, has significant implications in basic science and biomedicine. Mass spectrometry (MS)-based single-cell proteomics represents an emerging technology for system-wide, unbiased profiling of proteins in single cells. However, significant challenges remain in analyzing an extremely small amount of proteins collected from a single cell, as a proteome-wide amplification of proteins is not currently feasible. Here, we report an integrated spectral library-based single-cell proteomics (SLB-SCP) platform that is ultrasensitive and well suited for a large-scale analysis. To overcome the low MS/MS signal intensity intrinsically associated with a single-cell analysis, this approach takes an alternative approach by extracting a breadth of information that specifically defines the physicochemical characteristics of a peptide from MS1 spectra, including monoisotopic mass, isotopic distribution, and retention time (hydrophobicity), and uses a spectral library for proteomic identification. This conceptually unique MS platform, coupled with the DIRECT sample preparation method, enabled identification of more than 2000 proteins in a single cell to distinguish different proteome landscapes associated with cellular types and heterogeneity. We characterized individual normal and cancerous pancreatic ductal cells (HPDE and PANC-1, respectively) and demonstrated the substantial difference in the proteomes between HPDE and PANC-1 at the single-cell level. A significant upregulation of multiple protein networks in cancer hallmarks was identified in the PANC-1 cells, functionally discriminating the PANC-1 cells from the HPDE cells. This integrated platform can be built on high-resolution MS and widely accepted proteomic software, making it possible for community-wide applications.

## 1. Introduction

Direct measurement of unique constellations of proteins produced by single cells promises to expand our understanding of molecular cell-to-cell differences (heterogeneity) and their contribution to cellular functions in both disease and health. Single-cell proteomics can provide critical biological insight into the cellular heterogeneity that is masked by bulk scale analysis [1,2,3,4,5,6,7,8,9,10,11,12]. In contrast to antibody-based approaches, which are confined to measuring a small number of preselected proteins in a cell, mass spectrometry (MS)-based single-cell proteomics represents an emerging technology for system-wide, unbiased profiling of proteins in single cells or single cell clusters [13,14,15]. Since cell and tissue heterogeneity is a fundamental issue in many disease studies and proteins are the essential functional biomolecules that participate in all cellular physiologic processes, single-cell proteomics is highly relevant for various research in health and diseases. However, unlike DNAs and RNAs, there is no proteome-wide amplification approach currently available for proteins, posing great challenges in single-cell proteomics.

Mounting efforts have recently been made to improve MS-based single-cell proteomic technology, including isobaric labeling/carrier proteome-based approaches, such as SCoPE-MS [16,17], iBASIL [18], and SCeptre [19], as well as label-free approaches [7,20,21,22,23]. The application of the ion mobility technique has also been demonstrated to improve the sensitivity in single-cell proteomic analysis [24,25]. In addition, various single-cell sample preparation methods, such as iPAD [26], nanoPOTS [27], OAD [20], and microfluidic chip [23], have been introduced. These innovations and community efforts have greatly improved the sensitivity and utility of single-cell proteomics. For example, the utility of the carrier proteome, which is an isobaric labeled bulk sample added at 25×–500× amount to a single-cell proteome, has facilitated the protein identification in a single-cell proteomic analysis. Studies have reported over 2000 proteins being identified in a single-cell analysis with the aid of a carrier proteome [18,21]. While this multiplexed labeling-based approach has generated excitements in single-cell analysis, challenges and limitations have recently been indicated in association with the use of isobaric labeled carrier proteome [17,28]. As an emerging technology, single-cell proteomics is still at its early stage, lagging behind single-cell genomics and transcriptomics, which have been widely applied in routine analysis. One of the major challenges in dissemination and application of MS-based single-cell proteomics relies on the availability of a platform technology that is not only ultrasensitive, but also high-throughput, and can be practically adapted for various research applications.

Building upon the emerging concept of spectral library-based approach [29,30,31,32], we here report a unique single-cell proteomics platform that is conceptually different, highly sensitive, and well suited for large-scale analysis. We demonstrated that by using this spectral library-based single-cell proteomic platform, we could dissect the proteome of cell types and states with single-cell resolution to distinguish the difference between normal and cancerous pancreatic ductal cells with resolved cellular heterogeneity. The development of this platform technology may facilitate community-wide applications of single-cell proteomics for various studies, such as interrogation of tumor heterogeneity and analysis of circulating tumor cells.

## 2. Materials and Methods

Cell culture and pseudo single-cell samples. The PANC-1 pancreatic cancer cells (ATCC, Manassas, VA, USA) were maintained in DMEM (ATCC, Manassas, VA, USA) supplemented with 10% fetal bovine serum (FBS) and 1% penicillin–streptomycin. Immortalized normal human pancreatic ductal epithelial cells (HPDE-c7, gift from Dr. Xiaodong Cheng’s lab, UT Health Science Center at Houston, Houston, TX, USA) were maintained in keratinocyte serum-free medium supplemented with bovine pituitary extract and epidermal growth factor (Invitrogen, Carlsbad, CA, USA). After cells reached 70–80% confluence in culture flasks, they were detached with trypsin, washed twice with PBS, and collected for proteomic analysis.

In the method development, HeLa cell lysate digests mimicking the protein content of 1 cell (100 picograms), 5 cells (500 picograms), 10 cells (1 nanogram), 50 cells (5 nanograms), 100 cells (10 nanograms), and 500 cells (50 nanograms) were prepared.

DIRECT single-cell sample preparation. An “all-in-one” approach was developed for single-cell sample preparation by processing a single-cell sample directly in a screw cap LC injection vial (Thermo Fisher Scientific, Waltham, MA, USA). This single-cell protocol for direct LC–MS/MS analysis, namely, DIRECT, was simple and easy to perform, with no additional sample transferring. After separation, individual cells were picked under a microscope and transferred to each HPLC injection vial with 3 µL of PBS. Cells were subjected to freeze–thaw cycles for three times by placing the vials on dry ice and thawing briefly on ice. After each thaw cycle, cells were sonicated for 3 min in an ultrasonic cold-water bath (Fisher Scientific). Samples were reduced with 10 mM dithiothreitol (DTT) by adding 1 µL DTT stock solution (40 mM) and incubating for 1 h at 50 °C. The samples were alkylated with 25 mM iodoacetamide (IDA) by adding 1 µL IDA stock solution (125 mM) and incubating at room temperature for 30 min in the dark. A total of 1 µL of 350 mM ammonium bicarbonate was added to adjust the pH (≈7.8). The samples were digested with 1 ng of MS-grade trypsin (Thermo Fisher) at 37 °C overnight. In each step, the evaporated liquid that was accumulated at the back of the lid was collected by briefly centrifuging the glass vials placed in 50 mL tubes. The reaction was stopped, and samples were acidified by adding 1 µL of 5% formic acid. The final volume of a sample in an injection vial was ≈8 µL, and 5 µL of the sample was injected for LC–MS/MS analysis.

LC–MS/MS analysis. The tryptic digested samples were analyzed by LC–MS/MS using a Q Exactive™ HF-X Orbitrap™ mass spectrometer (Thermo Fisher Scientific) interfaced with an UltiMate 3000 Binary RSLCnano HPLC System. The samples were first loaded into a 5 mm trap column packed with 5 µM/100 Å C18 material (Thermo Fisher Scientific) using 98% buffer A (0.1% formic acid in water) and 2% buffer B (0.1% formic acid in acetonitrile) at a flow rate of 3 µL/min. The samples were separated in a self-packed C18 analytical column (100 µm × 30 cm) packed with 3 μm Reprosil-Pur C18-AQ material (Dr. Maisch, Ammerbuch, Germany) using a 90 min linear gradient from 2% to 35% buffer B versus buffer A at a flow rate of 350 nL/min. Mass spectrometric analysis was performed using the data-dependent acquisition (DDA) mode optimized for trace analysis. The survey scan was performed with 60,000 resolution from 400 to 1600 m/z with an AGC target of 3 × 10^6^ and a max injection time of 50 ms. Monoisotopic masses were then selected for fragmentation for the 25 most abundant precursor ions within a dynamic exclusion range of 15 s. The minimum intensity thresholds for bulk scale and single-cell analysis were set as 2.3 × 10^5^ and 2.0 × 10^4^, respectively. Fragmentation priority was given to the most intense ions. Precursor ions were isolated using the quadrupole with an isolation window of 1.6 m/z for bulk scale analysis and 2 m/z for single-cell analysis. Higher energy collisional dissociation (HCD) was applied with a normalized collision energy of 28%. MS/MS scans were carried out with a resolution of 7500. The minimum AGC target for MS/MS was set to 5.0 × 10^3^ and the maximum injection times were limited to as follows according to the sample amounts: 250 ms (100 pg to 1 ng), 100 ms (5 ng to 10 ng), and 22 ms for 1 µg.

Database search and construction of spectral library. The MS/MS data were searched against the UniProt human protein database (UniProt ID: UP000005640 (canonical), released June 2020; 20,394 protein entries) for peptide/protein identification using the Comet algorithm [33] embedded in the Trans-Proteomic Pipeline [34]. Carbamidomethylation of cysteine was set as fixed modification, and oxidation of methionine was set as variable modification. The peptide assignment was validated with PeptideProphet [35], and a probability score in correspondence with an error rate of 0.01 was applied to filter the peptides. The MS/MS data acquired from the bulk scale analysis (1 µg injection) were used to build the spectral library using the Skyline software [36]. The library used a PeptideProphet probability score in correspondence with an error rate of 0.01 as a cutoff for peptide selection. In addition, to ensure a more stringent identification for single-cell analysis, a “Targets” list was created, which only included peptides with an error rate of 0.01 and an E-value [37] ≤ 0.001. Based on the target-decoy database search results, these stringent criteria ensured a decoy-estimated false discovery rate (FDR) < 0.001, and that only peptides with a high-quality MS/MS spectrum were included in the “Targets” list for single-cell data analysis.

Spectral library based single-cell proteomic data analysis. The raw data of single-cell analysis were imported into Skyline and processed with the spectral library established by the bulk scale analysis. Peptide identification was made by spectral matching of accurate precursor mass, retention time, and isotopic distribution. The peptide settings were limited to peptides with 7–50 amino acids, differential modification of methionine oxidation, and fixed modification of cysteine carbamidomethylation. The transition settings were restricted to the peptides with charges of 2, 3, and 4. The first 3 isotopic peaks of a precursor ion were selected for the spectral matching with a mass tolerance of 0.055 m/z. The retention time window was set as 5 min. Peptides with an Isotope Dot Product (idotp—a measure of peak areas in comparison with the predicted isotope distribution based on the chemical formula for the peptide) less than 0.5 were excluded for further analysis. Peptide quantification was made at the MS1 level using the sum of the first 3 isotopic peaks. The abundance of each peptide was normalized to the total ion current (TIC) and presented as ion per million (IPM) using the following formula: Normalized Intensity (IPM) = Peptide Intensity/TIC × 1,000,000. Protein quantification was achieved by summation of the normalized intensities of the corresponding peptides. Proteins with an IPM < 0.01 were excluded in the comparison analysis. The median absolute deviation (MAD) method [38] was applied to evaluate the total intensity of each set of single-cell proteomic data for quality control. A dataset with the absolute value of modified Z score exceeding 2 was considered an “outlier” and would be removed from further analysis.

Statistical and enrichment analysis. Protein intensity data of the single-cell samples were subjected to nonlinear dimensional reduction and visualization using t-distributed stochastic neighbor embedding (t-SNE) [39]. Unpaired two-tailed Student’s *t*-test was used to compare the protein abundance in the single-cell samples between the HPDE and PANC-1. Proteins with a fold change ≥ 2.0 (PANC-1/HPDE ≥ 2.0 or ≤ 0.5) and a *p*-value ≤ 0.05 were selected for similarity and biological theme enrichment analysis. Similarity and t-SNE analyses were carried out using Morpheus (https://software.broadinstitute.org (accessed on 1 January 2022)) based on Pearson correlation. GO enrichment analysis of the generated datasets was performed using clusterProfiler 4.0 R package, and human gene annotation was from hallmark gene sets collected by the Molecular Signatures Database [40,41].

## 3. Results and Discussion

**Figure 1:**
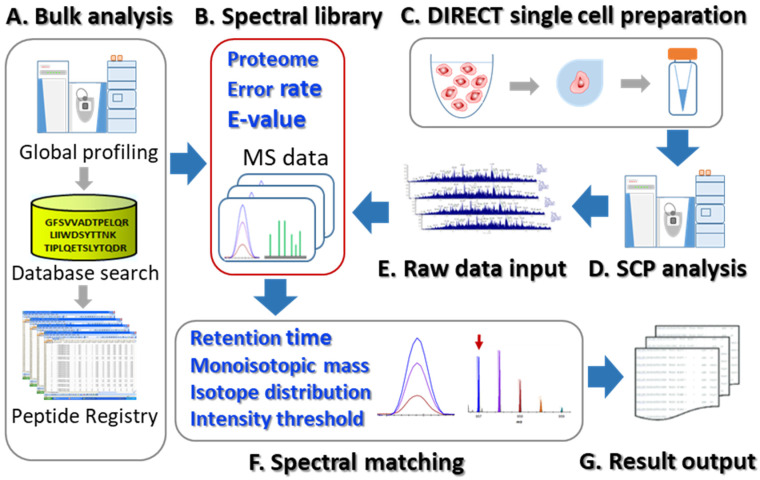
The workflow of the SLB-SCP platform. (**A**) Bulk scale analysis to acquire high-resolution data for library construction. (**B**) Construction of a spectral library with stringent identification criteria. (**C**) Single-cell sample preparation with the DIRECT protocol. (**D**) LC–MS/MS analysis optimized for single-cell proteomics. (**E**) Importing raw data for spectral library-based analysis. (**F**) Peak extraction through spectral matching with the spectral library. (**G**) Generating result output.

Spectral library-based single-cell proteomics. The spectral library-based single-cell proteomics (SLB-SCP) platform consists of the following modules: a high-resolution spectral library, an “all-in-one” single-cell DIRECT sample preparation process, LC–MS/MS analysis, and spectral library-based data analysis. The workflow is illustrated in Figure 1. First, high-quality, high-resolution data acquired from the bulk scale analysis were searched against a proteome database using stringent criteria for proteomic identification (Figure 1A). The confidently identified peptides were then used to construct a high-quality spectral library that serves as a registry for single-cell data analysis using spectral library matching (Figure 1B). Single cells were dispensed, isolated, and processed with the DIRECT protocol (Figure 1C), then analyzed with LC–MS/MS with the parameters optimized for traced proteomic analysis [42] (Figure 1D). The raw data of single cells were then matched against the spectral library for peptide identification based on monoisotopic mass, retention time, and isotopic distribution, and then filtered with an intensity threshold (Figure 1E, F). Peptide identification and intensity were then inferred for protein identification and quantification (Figure 1G).

In MS-based proteomics, it is well established that the fragmentation pattern of a peptide is specifically linked to its amino acid sequence and has been used as the essential mechanism for peptide and protein identification using database search. However, in a single-cell proteomic analysis, the low MS/MS signal intensity resulted from the extremely small amount of proteins available for an analysis hampers an effective proteomic identification using the conventional database search approach, or a data-independent acquisition (DIA)-based analysis. Alternatively, with high-resolution MS, a full breadth of information in MS1 spectra, including monoisotopic mass and isotopic distribution of a peptide precursor [43,44,45], along with its retention time (hydrophobicity), is accessible for peptide identification. These physicochemical properties are highly specific in association with the atomic composition and sequence of a peptide and could be used for peptide identification using a spectral library-based approach. Evolved from classic accurate mass and retention time (AMT) matching techniques for bulk scale analysis [46,47,48,49], the addition of peptide isotopic distribution as a critical criterion for peptide identification is essential for a single-cell analysis given the very low signal-to-noise ratio, as an isotopic distribution matching can explicitly distinguish a peptide precursor signal, which has a well-defined isotopic envelope, from the random noise for a precise peak extraction.

**Figure 2:**
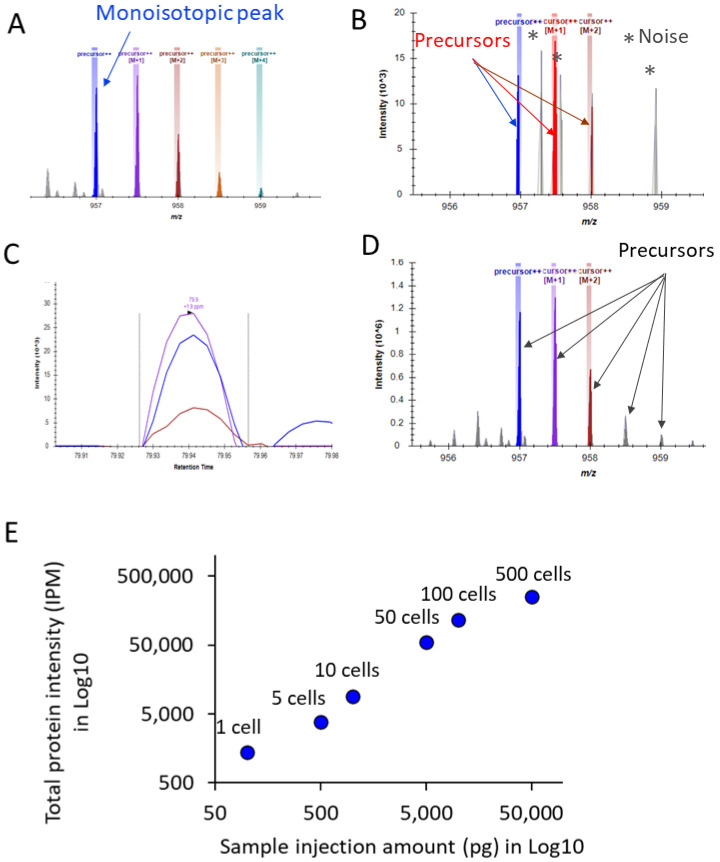
Exemplification of spectral matching and peak extraction using peptide LGGSLADSYLDEGFLLDK (CCT2) as an example. (**A**) Isotopic envelope of the peptide. (**B**) Isotopic distribution matching of the first 3 isotopic peaks detected in a pseudo single-cell sample (idotp score 0.77). The peaks in grey color are random noise. (**C**) Elution profile of the peptide detected in a pseudo single-cell sample. (**D**) Isotopic distribution matching of the first 3 isotopic peaks detected in a pseudo 500-cells sample (idotp score 0.99). (**E**) Correlation of the total normalized intensities of the proteins identified in the pseudo single-cell samples with the sample injection amount.

As an example, the isotopic distribution of peptide LGGSLADSYLDEGFLLDK derived from human T-complex protein 1 subunit beta (CCT2) is illustrated in Figure 2A. In the pseudo single-cell analysis of HeLa cell lysate (100 picograms), using the first three isotopic peaks for spectral matching based on monoisotopic mass, isotopic distribution, and retention time window, the precursor signals were identified from the random noise and accurately extracted from the MS1 spectra despite the low intensity (Figure 2B). With this data extraction approach, we could obtain the elution profile of this peptide for quantification (Figure 2C). To attest the accuracy of the precursor extraction, Figure 2D shows the detection of the isotopic envelope of the same peptide in a pseudo 500-cells sample (50,000 picograms), which had much higher signal-to-noise ratio, showing exactly the same spectral matching with the first three isotopic peaks, as we observed in the single-cell analysis (Figure 2B). Based on the proteins identified in the pseudo single-cell samples, the total normalized intensity of these proteins measured in the pseudo samples of 1, 5, 10, 50, 100, and 500 cells increased correlatedly (R = 0.96) with the amount of the samples injected for analysis, reflecting the overall accuracy in the precursor extraction (Figure 2E). Such a spectral library-based approach, which can be built on existing software, such as Skyline [36], affords a precise extraction of precursor signals enabling highly sensitive identification and quantification, thus allowing an in-depth, direct interrogation of the endogenous proteome of a single cell without the complications of using a carrier proteome [17,28,50].

**Figure 3:**
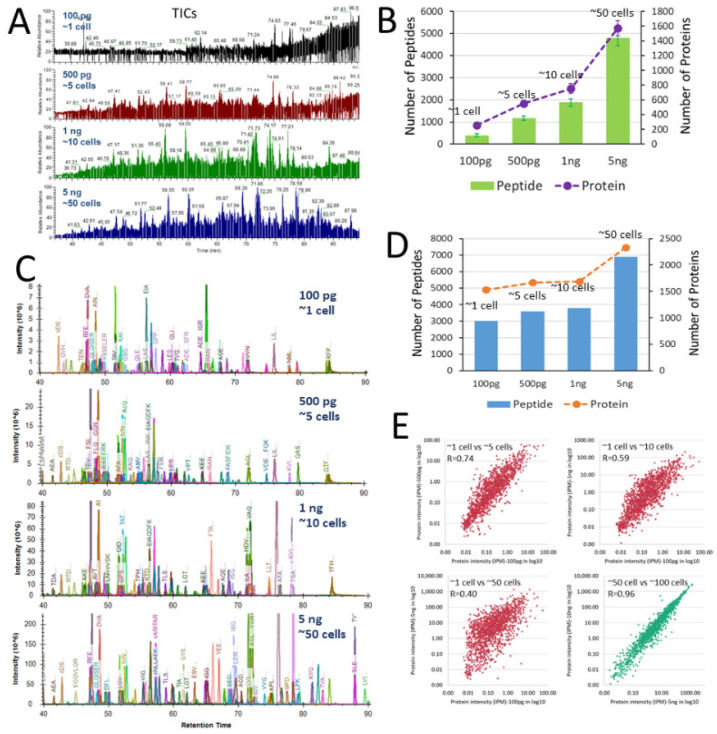
Assessment of single-cell analysis using SLB-SCP platform. (**A**) The raw total ion chromatograms (TICs) of HeLa cell digests for pseudo samples of a single cell (100 pg), 5 cells (500 pg), 10 cells (1 ng), and 50 cells (5 ng). (**B**) Number of unique peptides and proteins identified in the HeLa cell digests with 1% error rate according to a database search. (**C**) Extracted ion chromatograms (EICs) of the HeLa cell digests with SLB-SCP using the spectral library generated from the bulk scale analysis. (**D**) Number of unique peptides and proteins identified in all three replicates of the HeLa cell digests using SLB-SCP with stringent criteria (error rate: 1%, E-value ≤ 0.001, FDR < 0.001, idotp ≥ 0.5, IPM ≥ 0.01). (**E**) Correlations of the protein abundances of pseudo single-cell with pseudo 5, 10, and 50 cells (red plots), and correlation of the protein abundances of pseudo 50 cells with pseudo 100 cells (green plot). The data showed a non-linear dynamic range in protein abundances measured in the pseudo single cell in correlation with the bulk scale analysis. The plots are in log10 scale.

Assessment of SLB-SCP platform. To assess the SLB-SCP platform, HeLa cell lysate mimicking the protein content of 1 cell (100 picograms), 5 cells (500 picograms), 10 cells (1 nanogram), and 50 cells (5 nanograms) were prepared and interrogated using LC–MS/MS protocols optimized for traced proteomic analysis [42]. As exemplified in Figure 3A, the intensity of the total ion chromatograms (TIC) of the pseudo single-cell sample was very low. With a 1% error rate cutoff, the database search only identified ≈250 proteins from a pseudo single-cell sample, while the protein numbers identified increased with the protein amounts injected (Figure 3B). On the other hand, using the SLB-SCP approach with a spectral library generated from the bulk scale analysis of 1 µg of HeLa digests, we were able to extract the MS1 precursor ion signals from the TICs and show a significant increase in the signal-to-noise ratio of the peptides (Figure 3C). With additional criteria applied to peak intensity, on average, 2238 unique proteins were identified in a pseudo single-cell sample, including 1530 proteins identified in all three replicates (Figure 3D). Using the “ID-Pair” approach [51,52], we estimated that the false transfer rate (FTR) from the bulk library, which only included peptides identified with stringent criteria, to the single cell proteome was about 24% (≈76% overlap). The average number of the peptides identified using SLB-SCP was 12-fold compared to the number identified by the conventional database search, which was significantly compromised due to the low intensity in MS/MS spectra. The majority (91%) of the peptides identified from the pseudo single-cell sample by SLB-SCP had a monoisotopic mass deviation ≤ 20 ppm from the theoretical values (Figure S1A) and were well overlapped with the peptides (92%) and proteins (97%) identified in the pseudo 50-cells sample (Figure S1B).

Notably, protein intensities in pseudo single-cell samples correlated well with that in pseudo 5 cells samples, while such a correlation decreased as the protein amount increased, showing a correlation coefficients (R) of 0.74, 0.59, and 0.40 with the pseudo 5, 10, and 50 cells, respectively (red plots in Figure 3E). In contrast, protein intensities in pseudo 50 cells were well correlated with those in pseudo 100 cells (R = 0.98, green plots in Figure 3E). In correlation with the proteins measured in the bulk scale analysis (1 µg) for the library construction, as expected, the single-cell intensity data were poorly correlated with the data in bulk (Figure S2). These observations suggested that quantification from single-cell level to bulk scale was not linearly correlated, highlighting the importance of performing quantitative analysis at a single-cell level, as it may reflect different proteome landscapes that are not accessible from a bulk scale analysis.

**Figure 4:**
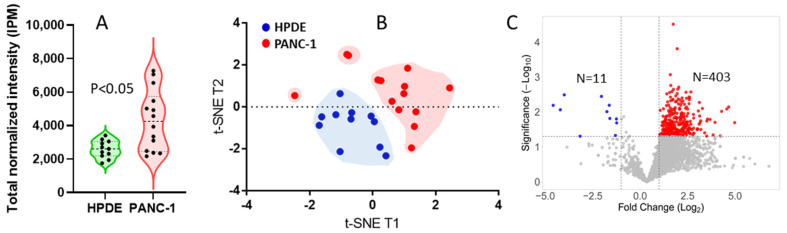
Single-cell analysis of HPDE and PANC-1 cells by SLB-SCP. (**A**) Comparison of total protein contents in the single cells of HPDE and PANC-1. (**B**) t-distributed stochastic neighbor-embedding (t-SNE) plot of the normal HPDE and cancerous PANC-1. While HPDE cells were aggregated, PANC-1 cells displayed 3 distinct subgroups. (**C**) Volcano plot of protein abundance ratio in comparison of PANC-1 with HPDE (PANC-1/HPDE).

Single-cell analysis of HPDE and PANC-1 cells in cell types and heterogeneity. We applied the SLB-SCP platform to analyze normal and cancerous pancreatic ductal cells (HPDE and PANC-1, respectively) at a single-cell level to further attest its robustness and applicability. Each individual cell was collected and processed for LC–MS/MS analysis directly in an injection vial using the DIRECT protocol, an “all-in-one” method for single-cell sample preparation (see the Materials and Methods section), with which the entire sample preparation process was performed in the HPLC injection vial with no sample transferring. This single-cell sample preparation method greatly reduces the sample loss and other potential contamination issues that are particularly critical to a single-cell proteomic analysis. While the DIRECT protocol was currently performed in a manual fashion, it is highly feasible to automate this technique for a high throughput analysis. With stringent criteria (error rate: 1%, E-value ≤ 0.001, FDR < 0.001, idotp ≥ 0.5, IPM ≥ 0.01) for peptide and protein identification, we compared 2632 proteins across 25 single-cell samples, including 11 HPDE and 14 PANC-1. Notably, the total proteome abundances in the single cells of HPDE group were highly similar, whereas the proteome contents in PANC-1 single cells were dispersed in a wide range and showed an overall higher abundance compared to HPDE (Figure 4A). The hierarchical clustering heatmap of protein abundances for all proteins identified in the single cells of HPDE and PANC-1 is provided in Figure S3. The t-SNE [39] plot of protein abundances in the single cells demonstrated a segregation of the 11 HPDE cells and the 14 PANC-1 cells (Figure 4B). While the HPDE cells were aggregated together, the PANC-1 cells displayed three distinct subgroups, consistent with the heterogeneous nature of cancer cells. These observations are consistent with the notion that cancer cells are more diverse compared to normal cells in part attributed to the dysregulated metabolism and cell biology driven by malignancy [53,54]. The volcano plot of protein abundance ratio in comparison of PANC-1 to HPDE showed that the majority of the differential proteins (*p*-value ≤ 0.05, fold-change ≥ 2.0) were overexpressed in the PANC-1 cancer cells (Figure 4C).

**Figure 5:**
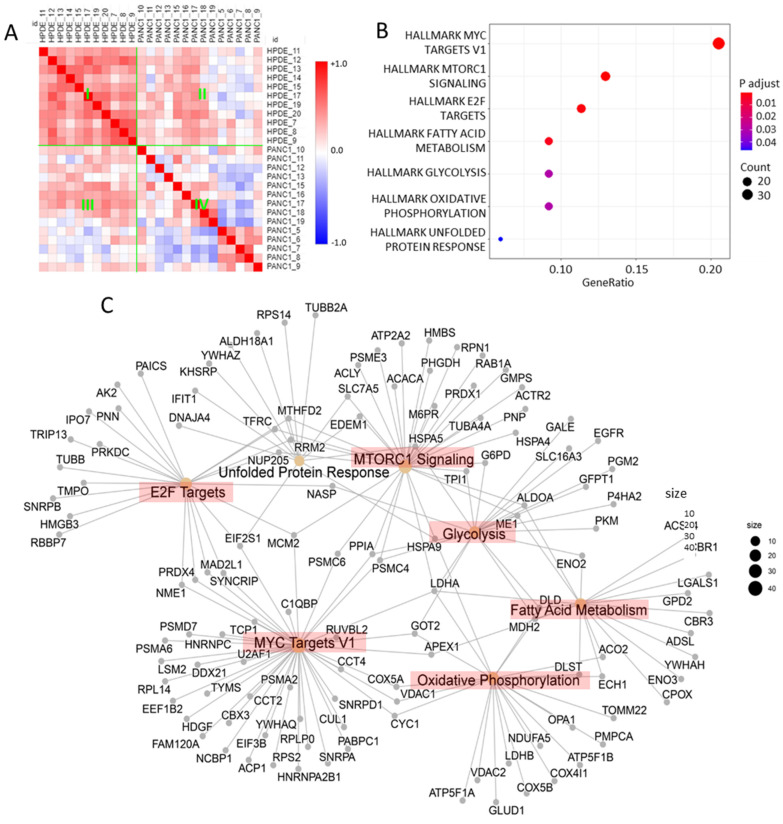
Similarity and functional analysis of the overexpressed proteins associated with PANC-1 cells. (**A**) Similarity matrix of the overexpressed proteins associated with PANC-1 based on Pearson correlation. The overexpressed proteins were highly correlated among the HPDE cells (Region I), whereas they were poorly correlated in the PANC-1 cells (Region IV) due to greater cellular heterogeneity. (**B**) Cancer hallmark enrichment analysis of the overexpressed proteins associated with PANC-1. (**C**) Network of the overexpressed proteins involved in the cancer hallmarks revealed.

Similarity and functional analysis of differential proteome associated with cancer cells. Similarity analysis indicated that the proteins overexpressed in PANC-1 were highly correlated among the HPDE cells, whereas they were poorly correlated in the PANC-1 cells (Figure 5A), confirming the observations in Figure 4. Functional analysis of the overexpressed proteins revealed that these proteins represented a significant upregulation of multiple pathways relevant to cancer hallmarks [53,55,56,57,58], including MYC, MTORC1, E2F, fatty acid metabolism, glycolysis, oxidative phosphorylation, and unfolded protein response (Figure 5B). The presence of these cancer hallmarks in PANC-1 functionally discriminated the PANC-1 cells from the HPDE cells at a single-cell level. The overexpressed proteins associated with these pathways closely interacted with each other group and formed a skeleton network (Figure 5C) that might, in part, underlie the proteome landscape for modulating the reprogrammed metabolism and dysregulated biology in PANC-1.

## 4. Conclusions

We reported on the initial development and utility of SLB-SCP, an integrated platform that effectively enhanced single-cell proteomic analysis by using a high-quality spectral library and a precursor ion extraction approach. In addition to accurate mass and retention time that correspond to monoisotopic mass and hydrophobicity of a peptide, the use of isotopic distribution, which is highly specific to the atomic composition of a peptide, adds an additional dimension for peptide identification. With the aid of the DIRECT sample preparation method, this ultrasensitive approach does not alter the endogenous proteome of a single cell with a carrier proteome, allowing label-free quantitative assessment of the proteins at the picogram level in individual cells, thus being well-suited for large scale analysis. This platform technology can be built upon widely accepted proteomic software, such as Trans-Proteomic Pipeline [34], MSFragger [59], and Skyline [36], and can acquire data for both unbiased discovery and targeted analysis, making it possible for community-wide applications in various research areas relevant to translational, pharmaceutical, and clinical applications. The application of SLB-SCP in characterizing individual cells of HPDE and PANC-1 demonstrated its potential to tackle the challenges in the interrogation of proteome and functional networks at the single-cell level, highlighting the unique significance of single-cell proteomics for functional analysis of the cellular heterogeneity. This study has opened an alternative opportunity using a conceptually different approach to enhance single-cell proteomic analysis. Challenges remain for further heightening its sensitivity and quantification, especially in automation of sample preparation, MS technology, and bioinformatics.

## Data Availability

The data are available on request from the corresponding authors.

## Supplementary Materials

The following supporting information can be downloaded at https://www.mdpi.com/article/10.3390/cells11152450/s1, Figure S1. Evaluation of the unique peptides and proteins identified in the pseudo single-cell by the SLB-SCP. Figure S2. Correlation plots of protein intensity data from a single cell (100 pg) and 500 cells (50 ng) vs. the bulk sample (1 µg). Figure S3. Hierarchical clustering heatmap of protein intensities (IPM) for all proteins identified in the single cells of HPDE and PANC-1.
