# Supplementary material for: Spectral Library-Based Single-Cell Proteomics Resolves Cellular Heterogeneity
Source: Cells. 2022 Aug 7;11(15):2450. doi: 10.3390/cells11152450 (PMC9368228; doi:10.3390/cells11152450)

Supplementary Figure S1. Evaluation of the unique peptides and proteins identified in the pseudo single-cell by the SLB-SCP. A) Distribution of the unique peptides identified in the single-cell samples (error rate: 1%, expect score ≤ 0.001, IPM ≥ 0.01, idotp ≥ 0.5) based on their mass deviation from the theoretical values. B) Comparison of the unique peptides and proteins identified in the pseudo single-cell samples with the pseudo 50-cells samples (bulk scale).

A

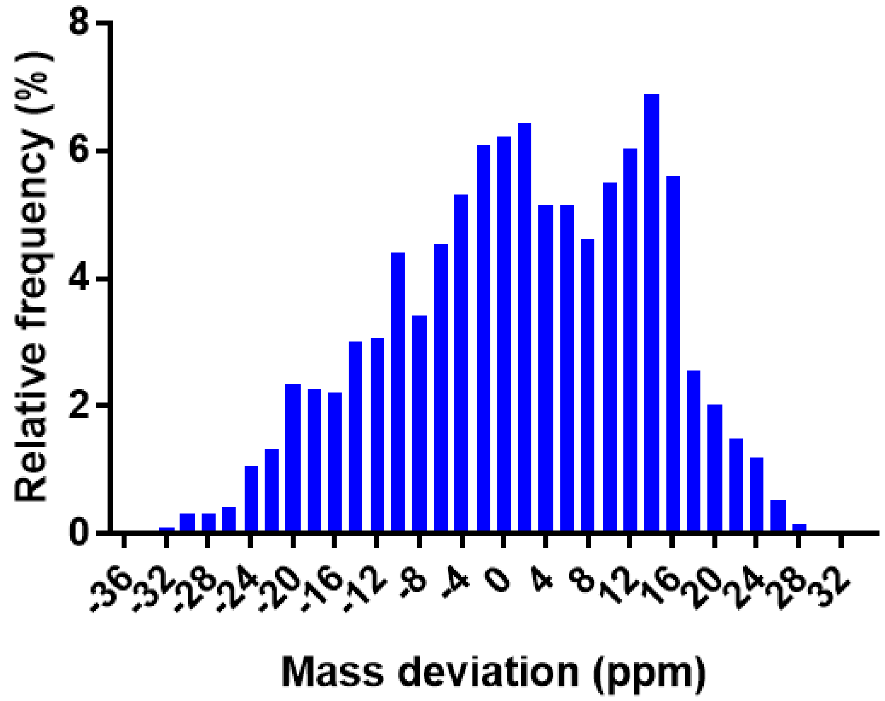

B

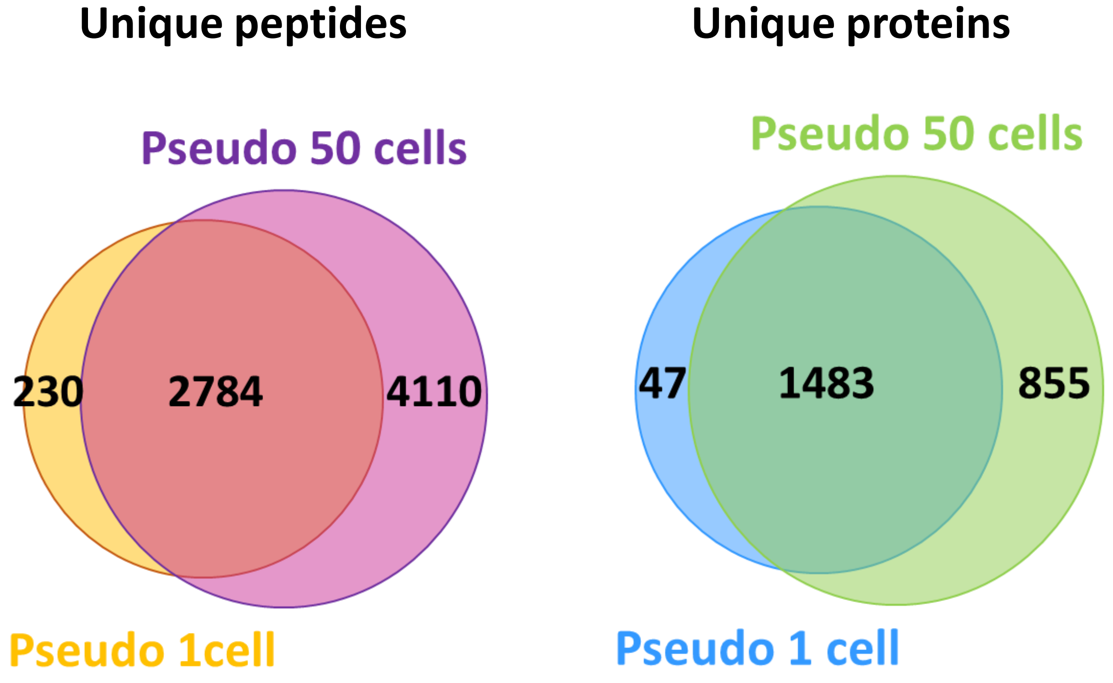

Supplementary Figure S2. Correlation plots of protein intensity data from single-cell (100 pg) and 500-cells (50 ng) vs the bulk sample (1 µg). A) Single-cell vs the bulk sample. B) 500-cells vs the bulk sample.

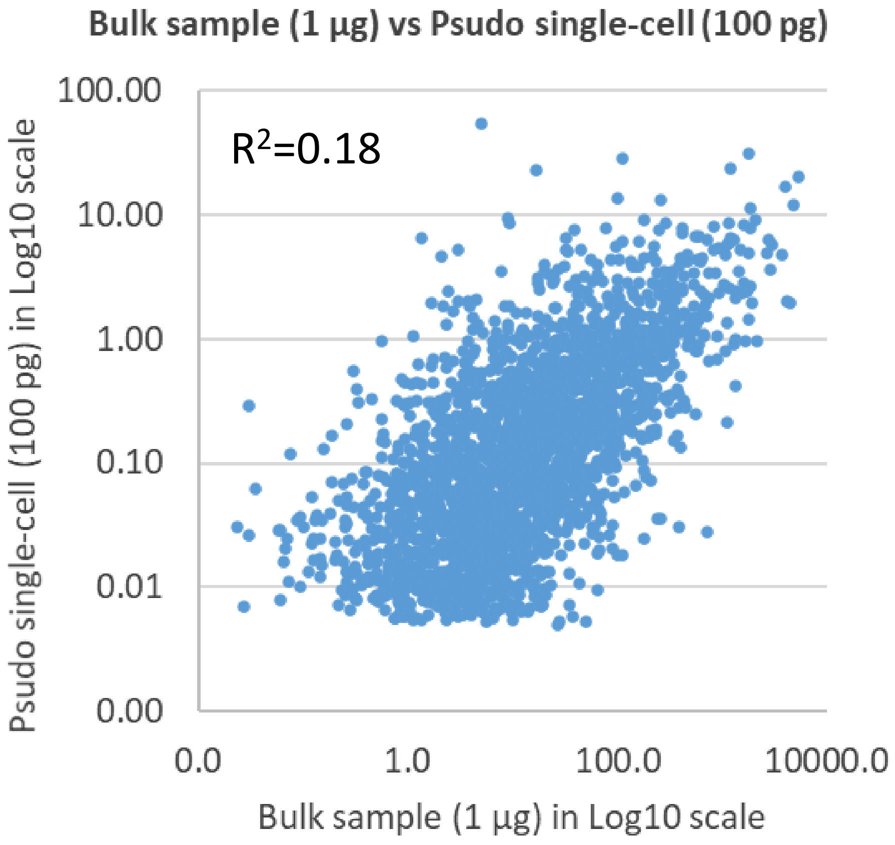

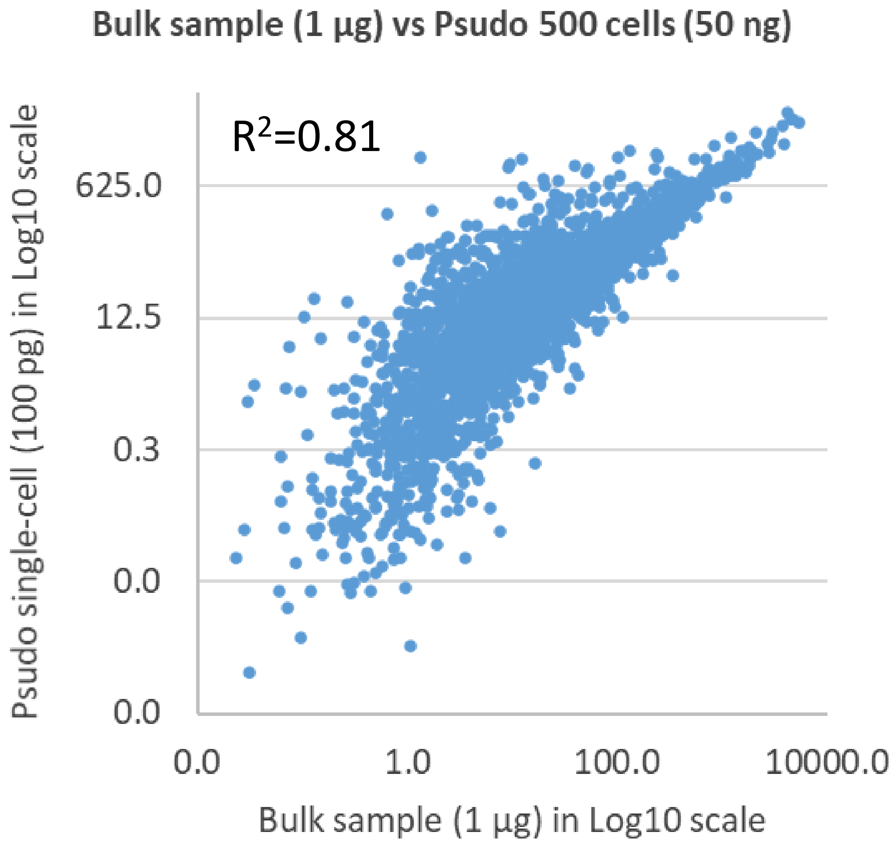

Supplementary Figure S3. Hierarchical clustering heatmap of protein intensities (IPM) for all proteins identified in the single cells of HPDE and PANC-1

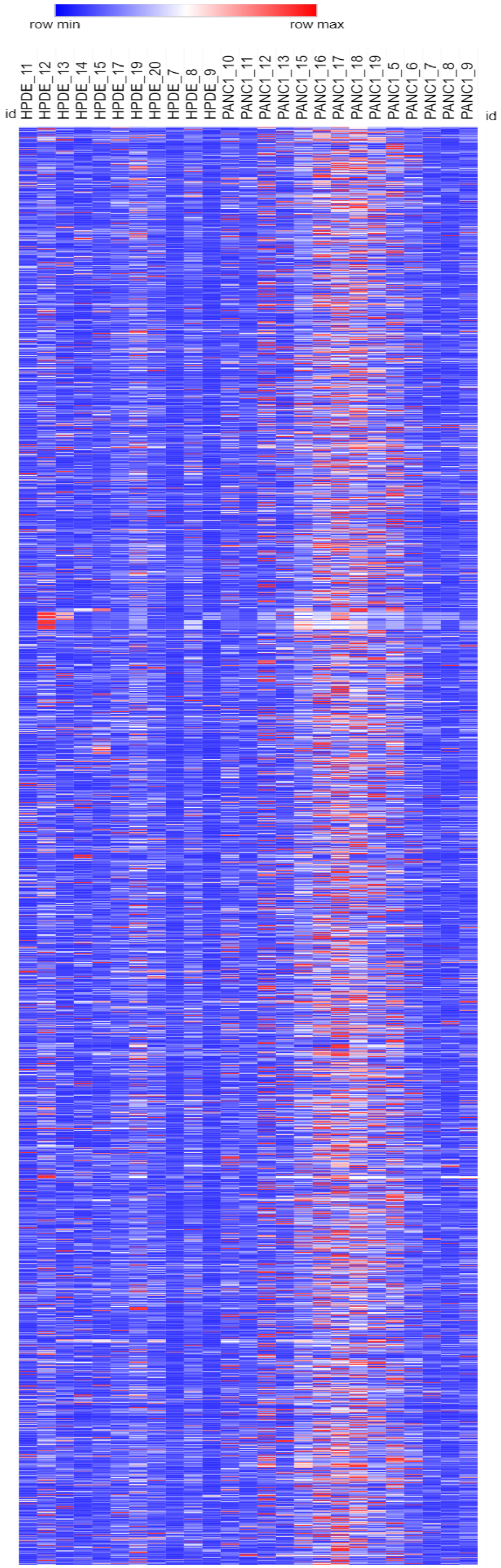

Supplement: Supplementary file 1 [file cells-11-02450-s001.zip › cells-1829220-supplementary.pdf]
